# MLC parameters from static fields to VMAT plans: an evaluation in a RT-dedicated MC environment (PRIMO)

**DOI:** 10.1186/s13014-019-1421-y

**Published:** 2019-12-02

**Authors:** Lucia Paganini, Giacomo Reggiori, Antonella Stravato, Valentina Palumbo, Pietro Mancosu, Francesca Lobefalo, Anna Gaudino, Antonella Fogliata, Marta Scorsetti, Stefano Tomatis

**Affiliations:** 1 Radiotherapy and Radiosurgery Department, Humanitas Clinical and Research Center, Rozzano, (Milan) Italy; 2 Department of Biomedical Sciences, Humanitas University, Pieve Emanuele, (Milan) Italy

## Abstract

**Background:**

PRIMO is a graphical environment based on PENELOPE Monte Carlo (MC) simulation of radiotherapy beams able to compute dose distribution in patients, from plans with different techniques. The dosimetric characteristics of an HD-120 MLC (Varian), simulated using PRIMO, were here compared with measurements, and also with Acuros calculations (in the Eclipse treatment planning system, Varian).

**Materials and methods:**

A 10 MV FFF beam from a Varian EDGE linac equipped with the HD-120 MLC was used for this work. Initially, the linac head was simulated inside PRIMO, and validated against measurements in a water phantom. Then, a series of different MLC patterns were established to assess the MLC dosimetric characteristics. Those tests included: *i)* static fields: output factors from MLC shaped fields (2 × 2 to 10 × 10 cm^2^), alternate open and closed leaf pattern, MLC transmitted dose; *ii)* dynamic fields: dosimetric leaf gap (DLG) evaluated with sweeping gaps, tongue and groove (TG) effect assessed with profiles across alternate open and closed leaves moving across the field. The doses in the different tests were simulated in PRIMO and then compared with EBT3 film measurements in solid water phantom, as well as with Acuros calculations. Finally, MC in PRIMO and Acuros were compared in some clinical cases, summarizing the clinical complexity in view of a possible use of PRIMO as an independent dose calculation check.

**Results:**

Static output factor MLC tests showed an agreement between MC calculated and measured OF of 0.5%. The dynamic tests presented DLG values of 0.033 ± 0.003 cm and 0.032 ± 0.006 cm for MC and measurements, respectively. Regarding the TG tests, a general agreement between the dose distributions of 1–2% was achieved, except for the extreme patterns (very small gaps/field sizes and high TG effect) were the agreement was about 4–5%. The analysis of the clinical cases, the Gamma agreement between MC in PRIMO and Acuros dose calculation in Eclipse was of 99.5 ± 0.2% for 3%/2 mm criteria of dose difference/distance to agreement.

**Conclusions:**

MC simulations in the PRIMO environment were in agreement with measurements for the HD-120 MLC in a 10 MV FFF beam from a Varian EDGE linac. This result allowed to consistently compare clinical cases, showing the possible use of PRIMO as an independent dose calculation check tool.

## Background

Accurate and fast calculation of a 3D dose distribution within the patient is one of the crucial procedures in the modern radiotherapy treatment planning systems and different kinds of algorithms have been developed through the years with this purpose.

Monte Carlo (MC) method is widely acknowledged to be able to estimate accurate dose distributions from radiotherapy beams generated by clinical linacs, and has been approached and used in radiotherapy during the last decades [1]. However, the need of long computation times has been a major obstacle to the use of MC in the clinical practice.

Clinical TPSs make use of algorithms with different levels of approximation; MC and Linear Boltzmann Transport Equation (LBTE) Solvers (e.g. Acuros) belong to the class that fully takes into account the electron transport in the medium. This class of algorithms allows the highest achievable accuracy in dose calculation [2].

PRIMO environment was developed to make a step forward towards the application of MC in radiotherapy treatment plan verification. PRIMO combines graphical user interface and a computational engine based on the MC code PENELOPE and the fast algorithm Dose Planning Method (DPM) [3]. It enables in particular the simulation of plans where intensity modulated (IMRT) and volumetric modulated arc therapy (VMAT) techniques are applied. It includes a library containing the main linac heads thus making it easier and faster to calculate the dose distribution for the desired beam [4]. These characteristics make PRIMO suitable to be used as an independent dose calculation verification since it has not the same beam model nor the same algorithm than the TPS [5]. However, a validation of the simulated beams is necessary. Some examples of phase-spaces validations against experimental measurements are available in literature [6, 7].

An adequate modeling of the multi-leaf collimator (MLC) is essential for accurate dose calculations in IMRT and VMAT treatment plans [8, 9]. Modern TPSs take into account in different ways the MLC characteristics such as leaf end design (or curvature), intra-leaf and inter-leaf transmission and tongue-and-groove effect. MLC modeling has to be checked during the TPS commissioning, and, similarly, this should be done also for MC implementations.

The modeling of the Varian HD-120 MLC (High Definition Multileaf Collimator) has been studied and described in literature by different authors using different MC systems [10, 11]. These works have been carried out for different beam energies and different linac models. In PRIMO the MLC geometries of specific linacs are stored in a library and cannot be modified by the user. However, a validation of a secondary collimation system as MLC is fundamental to allow using PRIMO as an external dose verification system.

A 10 MV flattening filter free (FFF) beam from an EDGE linac (Varian Medical Systems, Palo Alto, CA) was here studied since it is the most used for Stereotactic Body Radiation Therapy (SBRT) and Stereotactic Radiosurgery (SRS) treatments in our clinical practice [12, 13]. Aim of this work was to investigate the suitability of MC in PRIMO to produce accurate dosimetric results, by comparing MC simulations against measurements and Acuros calculation, with a particular focus on the MLC management.

Finally, MC in PRIMO and Acuros were compared in 5 clinical cases, summarizing the clinical complexity in view of a possible use of PRIMO for dose calculation independent check.

## Materials and methods

For this work a 10 MV FFF beam from a Varian EDGE linac equipped with the HD-120 MLC was used.

In a first phase of the work, the linac head was simulated in PRIMO, and tested on a phantom against measurements in water. Then, once assessed this initial phase, a series of MLC patterns were considered and the related dose distribution was simulated in PRIMO and measured with films in a solid water phantom, as well as evaluated with Acuros calculations. A second part of the study compared MC and Acuros calculations on clinical cases. Here below the details follow.

### PRIMO environment

For this work, PRIMO [14] was used from version 0.1.3.137 to 1.0.0.1756-beta following the software development updates.

PRIMO combines a graphical user interface with a general-purpose radiation transport code, PENELOPE, and the fast Dose Planning Method DPM algorithm [15, 16], specifically implemented in PRIMO for the simulation of radiotherapy beams. A complete simulation in PRIMO is divided into three segments: for the first one, S1, PRIMO allows the user to select the linac head of interest from a predefined geometry library, to tune the primary beam parameters, and perform the simulation of the upper part of the linac head. The output of S1 is a phase-space file representing the beam above the jaws. The second segment, S2, identifies the phase-space at the downstream end of the region corresponding to the bottom of the collimating devices. It includes the simulation of the secondary collimating system (both jaws and MLC). The output of S2 is a phase-space file representing the beam arranged for a specific plan, located at the bottom of the collimation system. Finally, the third segment, S3, estimates the absorbed dose within a phantom or a patient CT.

### Linac head simulation and phase-space validation

A phase-space of the S1 segment of our beam was simulated in PRIMO, using PENEASY/PENELOPE as simulation engine. For the linac head, an approximate empirical geometry named FakeBeam, developed by the PRIMO authors [17], was used with a 10 MV FFF beam. The primary beam was characterized by the following beam parameters for the initial electron beam: mean energy of 10.8 MeV, energy full–width at half–maximum (FWHM) 0, focal spot FWHM 0.1 cm, and beam divergence 0. Those are the default parameters suggested in PRIMO. The splitting roulette, a variance reduction technique described in [18] used in this work. A total number of 77 × 10^6^ histories were used for the simulation and a phase-space file (PSF) of 56 Gigabyte was obtained in segment S1. This PSF was used as the source of particles for the S2 and S3 segments, simulated together using DPM. In S2, the HD-120 MLC was selected when defining the field or importing the treatment plan (leaf geometry, to our best knowledge, is included in PRIMO, according to the manufacturers’ blueprints). During the S3 simulation, the transport parameters for the DPM included cut-off energies of 50 keV for photons and 200 keV for electrons. The S3 used, depending on the test case, water phantom, solid water phantom, or patient CT dataset. In order to reduce the statistical uncertainty, a splitting factor was applied for the S3 simulation as described in PRIMO User’s Manual [19].

PRIMO reports the average statistical uncertainty of the simulation considering all the voxels (voxel size for all the simulations was 1.5 mm in each direction) receiving more than 50% of the maximum absorbed dose and are given at 2 standard deviations. The variance reductions applied in S1 and S3 allowed obtaining uncertainties lower than 2%, except for tests with very small field sizes (5 mm), where a 3% value was accepted.

The validation of the PSF from S1 simulation was conducted against measurements, with static square fields shaped by the jaws, and not the MLC. Depth dose curves (PDD), profiles and OF at isocenter, 5 cm depth, were compared for different field sizes (2, 3, 5, 10 and 20 cm^2^). Measurements were acquired in a water phantom with the microdiamond detector (PTW).

The PSF obtained in the validation phase was used as the source of particles in all the simulations carried out in the tests described below

### MLC tests

The Varian 120-HD MLC has the 32 central leaf pairs with a 2.5 mm width at the isocenter, and the remaining have a width of 5 mm, to cover a field 22 cm long. To reduce the interleaf leakage, leaf sides are designed with a ‘tongue-and-groove’ arrangement, where dovetails shape the complementary tongue or groove regions of adjacent leaves. This structure reduces the interleaf fluence when the leaf sides are exposed to the radiation beam. This fluence reduction is known as tongue-and-groove (TG) effect [20] and can lead to under-dosages [21]. All leaf ends have a rounded edge design to minimize the penumbra variation for all leaves positions.

Both the static and the dynamic behavior of the MLC were investigated in two sets of tests summarized in Table 1 and described below.

**Table 1:** Tests for static and dynamic MLC used for this work. Main geometrical characteristics and relative measurements are reported

	Test	Jaws (cm)	MLC	Measurements	Analysis
Static	*MLC_square*	10 × 10	Square 2,3,5 and 10 cm side	microDiamond	Point dose
	*MLC_transm*	10 × 10	Fully closed on the central axis	Film	Profiles
	*MLC_alternate*	10 × 20	Bank B: even leaves open, odd leaves closed. Bank A: all closed	Film	Profiles
Dynamic	DLG-test	10 × 10	Gap: 2,4,6,10,14,16, 20 mm moving from left to right Transmitted dose (bank A closed and then bank B closed)	microDiamond	Point dose
	a-SG	10 × 22	Gap: 5, 10, 20, 30 mm TG: 0, 1	Film	Profiles
	a-OSG	6 × 8	Gap: 10, 30 mm TG: 0, 1	Film	Profiles

### Static MLC tests

#### *MLC_square*

MLC-defined square fields of 10 × 10, 5 × 5, 3 × 3 and 2 × 2 cm^2^, with the jaws set to 10 × 10 cm^2^ in all cases. For each field, the output factors, OFs (ratio between the dose of the test field and the open 10 × 10 cm^2^ field, for fixed MU) were evaluated in water.

#### *MLC_transm*

The MLC transmission was estimated with a field having the jaws set to 10 × 10 cm^2^, and with the MLC fully closed on the central axis. Dose profiles in the direction parallel to the leaf motion, at 0.1 cm (under a leaf), 5 cm depth, were analyzed in solid water phantom.

#### *MLC_alternate*

**Fig. 1:**
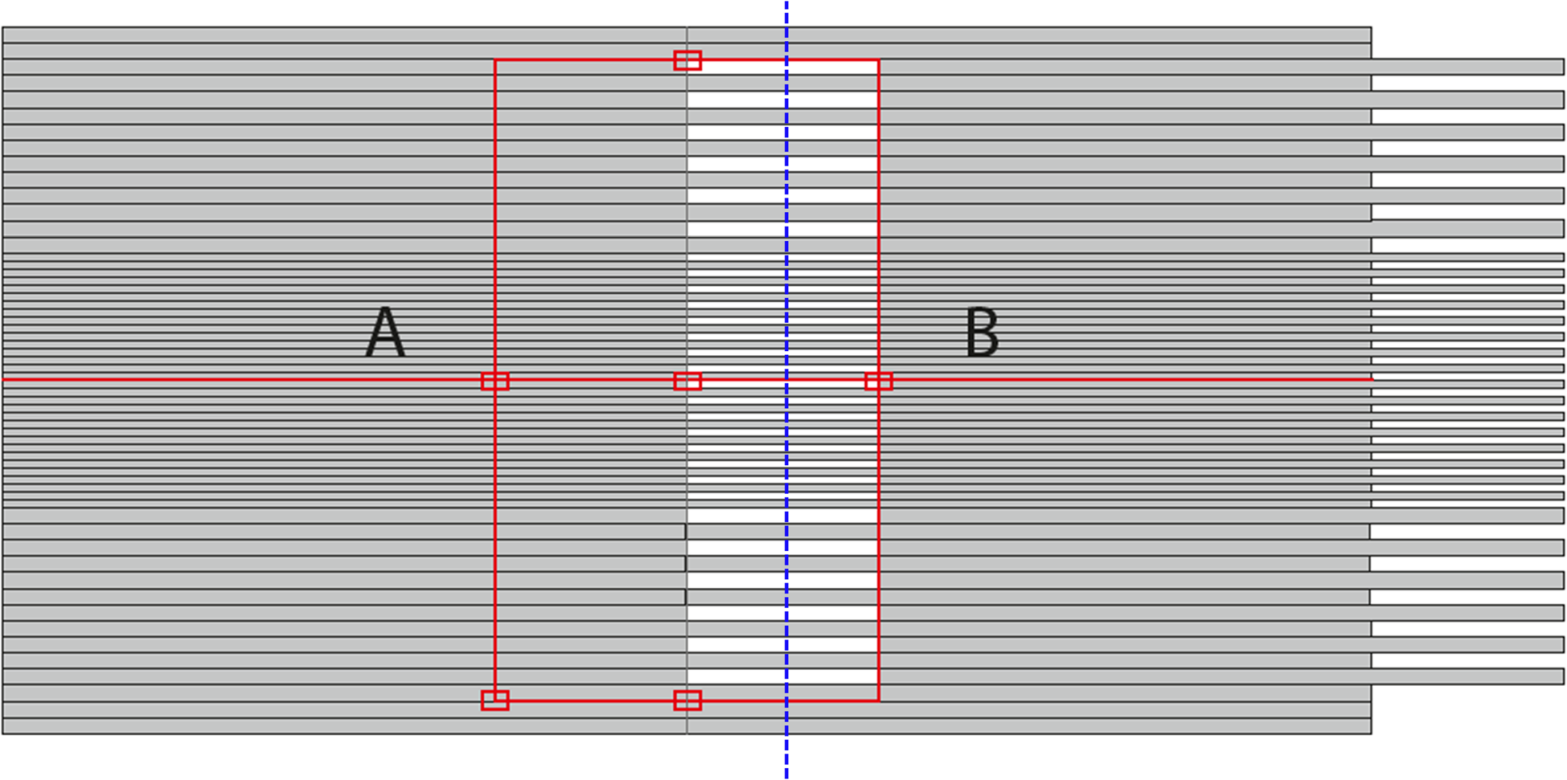
Configuration for the MLC bank **a** and bank **b** in the MLC_alternate test

A static field as suggested by Bergman et al. [22] was evaluated: the even MLC leaves of Bank B were set open while the odd-numbered leaves were closed in order to create a comb pattern; the Bank A leaves were all closed (see Fig. 1). A profile perpendicular to the leaf motion 2.5 cm off-axis under the comb pattern was analyzed, for both the 2.5 and the 5 mm leaf regions.

### Tests with dynamic MLC

The dynamic behavior of the MLC was tested at the leaf ends with a dosimetric leaf gap (DLG), and at the leaf side with the tongue-and-groove (TG) effect. The tests were repeated with MC simulations in PRIMO, with measurements with films, and with Acuros calculations in Eclipse.

#### *DLG-test*

DLG mimics the field size generated by closed opposed leaves (it is the FWHM of the leaf-end transmission peak). DLG was determined following the procedure reported by [23]. The doses delivered on the central axis, at isocenter at 5 cm depth, by dynamic fields with increasing sliding gaps were obtained. The gaps ranged from 2 to 20 mm, and the leaves moved from − 60 mm to + 60 mm with constant speed, resulting in uniform fluence within the field size set by the jaws to 10 × 10 cm^2^. The MLC transmission (average of the Bank A and B transmissions) was subtracted from the dynamic sliding gap field doses to obtain the corrected delivered dose per each gap. A linear relationship is determined between the corrected delivered doses and the corresponding gap width. The DLG is defined as the gap corresponding to the zero dose.

#### *Asynchronous sweeping gap (a-SG)*

**Fig. 2:**
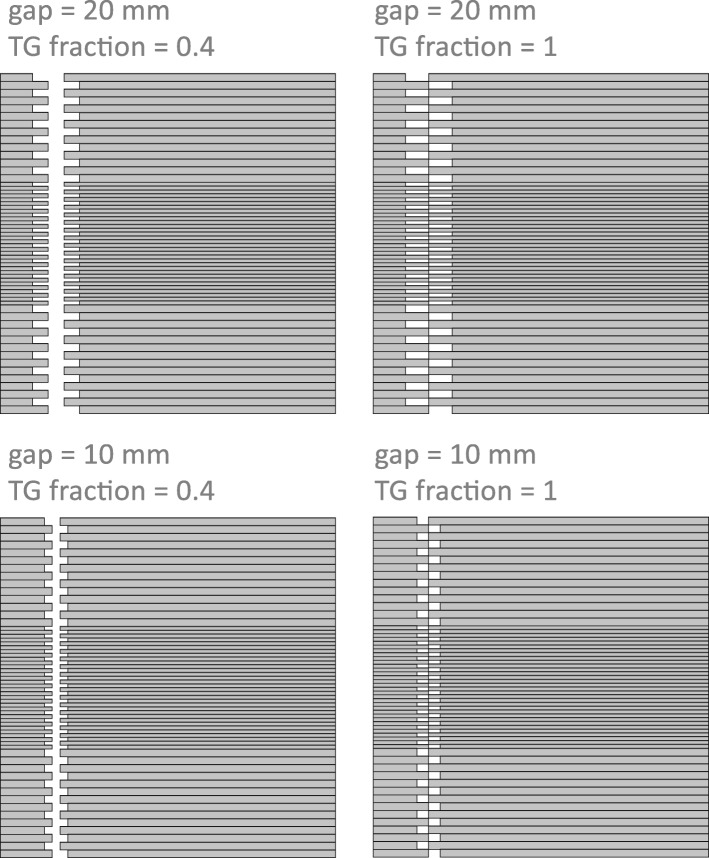
Example of MLC configuration in four of the a-SG tests where it is possible to see different gap values and different TG fractions [15]

This test is described in [24] to evaluate the effect of TG in IMRT fields. It is a ‘moving fence pattern’ with all leaves with even numbers shifted with respect to their neighbor leaves, generating a fence-shaped MLC pattern. All leaves move at the same constant speed, keeping the MLC pattern unchanged. All leaf pairs produce the same gap size, but, since leaves are not uniformly extended, this test incorporates the TG effect. For a fixed gap size (*g*) we can have different shifts between adjacent leaves (*s*) that determine different TG fractions defined as *TG fraction = s/g* (Fig. 2). Dose profiles were analyzed for sweeping gap values of 5, 10, 20 and 30 mm for different TG fractions (0, 0.4 and 1) at 10 cm depth in phantom. The original plans, optimized in Eclipse by Hernandez [24], have 29 control points that can be increased in PRIMO by a given integer factor [19] thus increasing the time resolution of dynamic plans. The new number of control points is calculated in PRIMO by linear interpolating the MLC leaves positions and the dose fraction ensuring a minimum of 200 control points for the plans used in this work.

#### *Asynchronous oscillating sweeping gap (a-OSG)*

This test is described in [24] to evaluate the effect of TG in VMAT arcs. A uniform MLC gap (of 10 and 30 mm in two tests) repeatedly moves across the field width at a constant speed during a full gantry rotation, generating an approximately uniform dose distribution in a cylindrical volume. Two TG fractions, 0 (aligned adjacent leaves) and 1 (shift between leaves equal to gap between two facing leaves), were used. The test analyzed dose profiles at 10 cm depth in a solid water phantom.

### Measurements of MLC tests

Point dose measurements (MLC_square and DLG_test) were acquired with a microDiamond detector (PTW, Freiburg, Germany, 2.2 mm radius chips) in an BluePhantom^2^ (IBA Dosimetry) water tank.

Profile measurements (Table 1) were acquired with films in a Plastic Water phantom (MULTIcube, IBA Dosimetry).

Radiochromic EBT3 films (GafChromic, ISP Technology, Wayne, NJ) were used, calibrated with the dose-exposure curve [25]. Calibration was performed in the range 0–5 Gy (0.25 Gy spacing between 0 and 1.25 Gy, and 1 Gy between 2 and 5 Gy). The films were scanned on the green channel of a 48-bit scanner (Epson Expression 1000XL, Epson America, Sunnyvale, CA) with a resolution of 72 dpi (pixel resolution of less than 0.4 mm). The films were placed in the scanner with accurate and reproducible procedure and orientation to exclude variations in the scanner response over scan field. The calibration curve was fitted with a third grade polynomial function using the OmniPro-I’mRT software (IBA Dosimetry). The uncertainty of the film measurements in the dose range of interest in this work can be considered < 3% [26].

### Acuros dose calculation in Eclipse TPS

MLC tests were repeated on the Eclipse TPS, and calculated with Acuros dose calculation algorithm. It is a linear Boltzmann transport equation solver, expected to have similar degree of accuracy of a MC simulation.

Regarding the MLC modeling, Eclipse considers a single MLC transmission value, input by the user during the beam configuration. This neglects the transmission modifications due to energy spectrum variations in the field area, or variations between leaves of different widths, or variations of the transmission with depth. The TG is modeled separately by modifying the fluence, extending the leaf projection in the direction perpendicular to the leaf motion by a fixed parameter [24, 27]. The rounded leaf ends are modeled through the DLG as described above. This parameter is used in Eclipse modifying the fluence, as generated by shifting the leaf end position back by half of the DLG value.

The MLC parameters used in Eclipse for the Acuros configuration in this work (for the 10 MV FFF beam) were: MLC transmission of 1.3%, and DLG equal to 0.41 mm.

All the above described tests were computed with Acuros in the same conditions for subsequent comparisons, using a dose calculation grid size of 1.5 mm.

### Clinical VMAT cases

For the last phase of this work, 5 patients were selected from the institutional database, covering a wide range of target volumes (from 0.9 to 995 cm^3^) and plan modulations (evaluated in terms of mean segmented opening and mean segmented area) in different anatomical regions (brain, lung and breast).

The treatment plans were optimized for VMAT technique in Eclipse, using the PO (Photon Optimizer) algorithm in its version 13.5, with an optimization resolution setting of 2.5 mm. The final dose distribution was calculated with Acuros using a grid size of 1.5 mm.

Acuros calculates the energy dependent electron fluence, based on the patient material properties derived from the Hounsfield Units (HU) of the CT dataset. For each material the specific chemical elemental composition is based on the ICRP Report 23 [28] and ICRP Report 89 [29, 30].

The DICOM files (plan, structures and CT images) were exported from Eclipse and then imported in PRIMO. The dose distributions in the patients, for each plan were simulated with the DPM using a voxel size of 1.5 mm. The medium material is assigned according to the material conversion, as reported in Table 2.

**Table 2:** HU and mass density ranges used in PRIMO and Acuros computations

Material	PRIMO HU range	PRIMO mass density range (g/cm^3^)	Acuros HU range	Acuros mass density range (g/cm^3^)
Air	− 1000, − 957	0, 0.0204	−1000, − 957	0, 0.0204
Lung	−957, − 400	0.0204, 0.594	− 967, − 374	0.0104, 0.624
Adipose Tissue	− 400, − 59	0.594, 0.969	− 434, 1	0.551, 1.001
Muscle, Skeletal	− 59, 88	0.969, 1.075	−59, 117	0.969, 1.093
Cartilage	88, 298	1.075, 1.199	57, 971	1.056, 1.600
Bone	298, 2832	1.199, 2.833	128, 2832	1.100, 2.830

The dose distributions obtained with PRIMO and Acuros, both reported as dose to medium, were compared in terms of 3D gamma analysis within the external patient contouring (3%-2 mm and 2%-2 mm) [31], using the tool implemented in PRIMO.

## Results and discussion

### Linac head simulation and-phase space validation

The simulations of static jaw-defined fields were compared with microdiamond measurements for 2, 3, 5, 10 and 20 cm^2^ squared fields.

The average point-by-point differences between measured and calculated PDDs were < 1% for fields ≥3 × 3 cm^2^. This result is in line with the one obtained by Hermida-López et al. [1] where the agreement between dose simulated with PRIMO and measurements was within 1.3%. Dose profiles showed average point-by-point differences below 2% for all considered field sizes. These results are in line to those obtained by Belosi et al. [6] for the Varian provided PSF for FFF beams validated with PRIMO.

Agreement between OF are within 0.4% down to the 3 × 3 cm^2^ field. For the 2 × 2 cm^2^ field the differences were found to be up to 1.1%. The current method to convert eV/g to Gy/MU in PRIMO does not correct for the radiation backscattered into the monitor chamber, which depends on the field size, particularly for small fields. As reported by Zavgorodni [32] the backscatter correction factor BSF, however, is small for the considered field sizes, with values of 0.2% for the 2 × 2 cm^2^ and negligible for larger fields. Correcting the simulated output of the 2 × 2 cm^2^ field by this BSF from a similar linac, the difference with measurements states below 1%.

The comparisons between MC and measurements showed that the PSF generated in PRIMO agrees with the 10 MV FFF beam from our EDGE linac, and further tests can be carried out. A deeper presentation of the results of the phase space validation, however, exceeds the aim of this paper.

### MLC tests

#### Static MLC tests

##### MLC_square

Calculated OF (MC in PRIMO and Acuros in Eclipse) for the static square MLC-defined fields were compared against measurements. The maximum difference was for the 2 × 2 cm^2^ field, of 0.5% for PRIMO, and 1.2% for Acuros, as shown in Table 3.

**Table 3:** OF relative to the MLC_square test

Field size (cm^2^)	Measured OF	PRIMO OF (diff.)	Acuros OF (diff.)
2 × 2	0.852	0.857 (0.5%)	0.862 (1.2%)
3 × 3	0.902	0.905 (0.3%)	0.907 (0.6%)
5 × 5	0.946	0.944 (−0.3%)	0.947 (0.0%)

##### MLC_transm

**Fig. 3:**
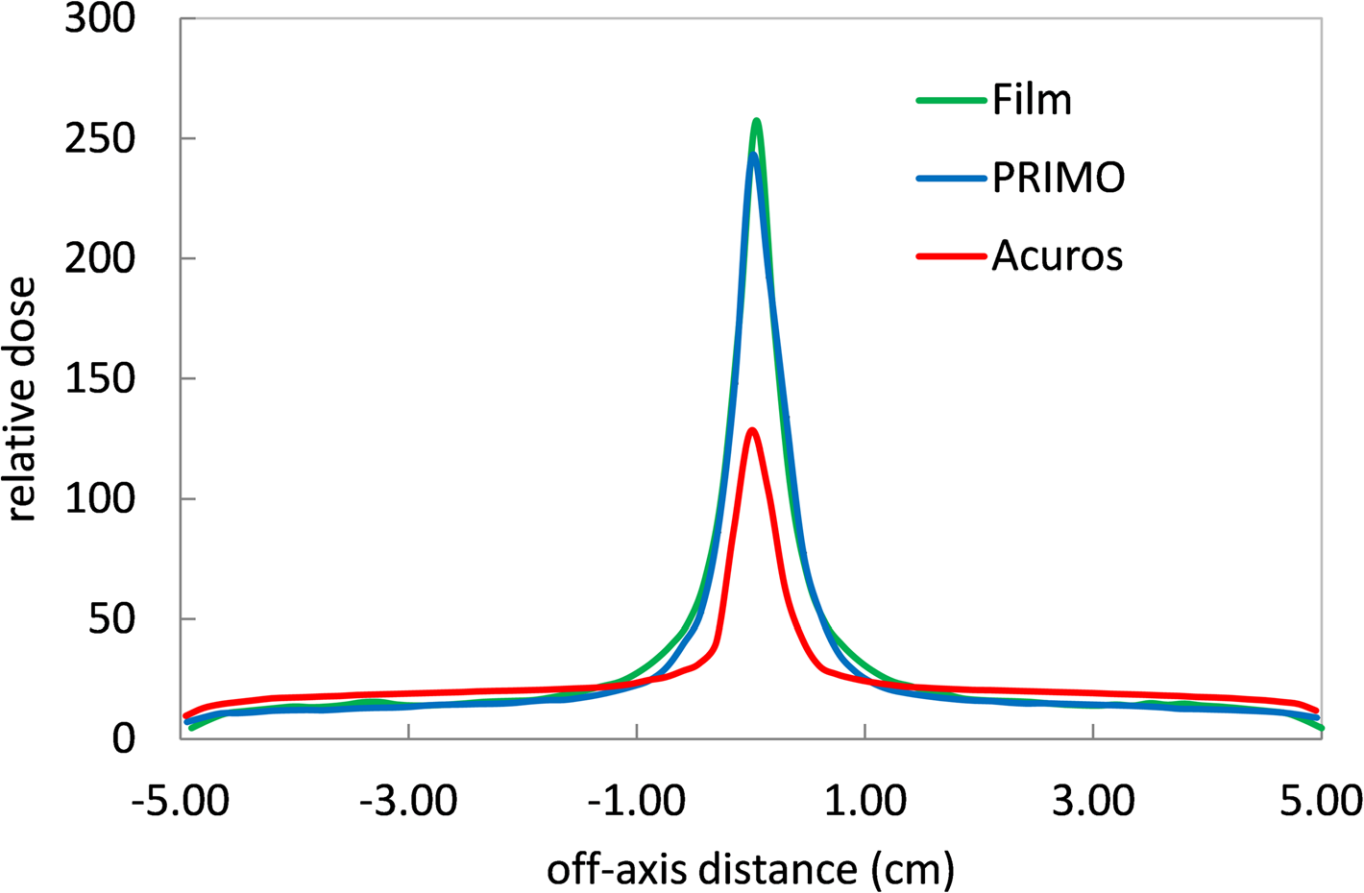
Profiles for the MLC_trasm test extracted under a leaf at 0.1 cm parallel to the leaves direction

Figure 3 reports the profiles parallel to the leaf motion, at mid-width of a 2.5 mm leaf. The peak evaluates the leaf end transmission. The relative discrepancy between PRIMO and film measurements is − 6%, result that is in agreement with what observed by Tyagi et al. [33], while Acuros underestimate the dose of about a factor 2. To note, all the data were rebinned at 1.5 mm, inducing a possible smoothing of the real profile.

The dose transmitted under the leaves was evaluated from the same profiles, at 2.5 cm off-axis. The transmission was 0.9, 1 and 1.3% for MC in PRIMO, film measurements and Acuros, respectively. This is in agreement with what observed in other works [24, 34]. The Acuros result equals the value of the MLC transmission set for the algorithm configuration, based on point measurements, including also some interleaf leakage, leading possibly to an overestimated value.

##### MLC_alternate

**Fig. 4:**
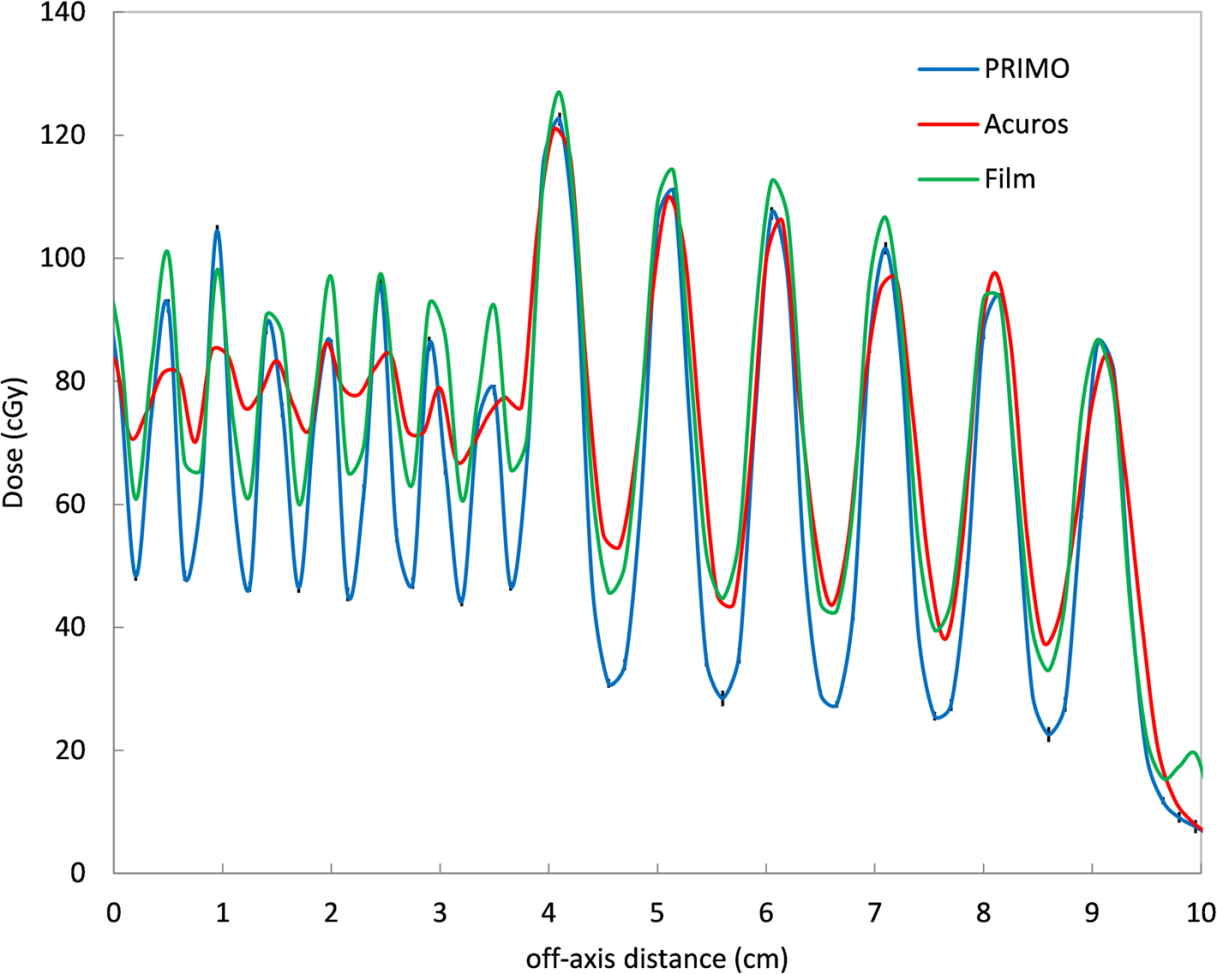
Dose profiles for the MLC_alternate test for the dose distributions obtained with PRIMO (blue), film measurements (green) and Acuros (red). All profiles are plotted in absolute dose

Figure 4 shows the profiles as shown with the blue dashed line of Fig. 1. The profiles present alternate peaks and valleys in correspondence of the open and closed leaves and the slightly peaked overall dose profile is due to the characteristic intensity distribution of the FFF beams, peaked on the central axis. MC data presented deeper valleys (lower dose under leaves) relative to the measurements (up to 32%) for the 2.5 mm leaves. On average the differences between MC and measurements are 13% under thin leaves and 15% under thick leaves, in line with the tendency found in the MLC_trasm test. Tyagi et al. [33] in their work reported an agreement of 1%, obtained with a Varian 120 leaf MLC and 6 MV energy. Bergman et al. [22], using MLC and beam quality very similar to ours, obtained discrepancies of 20–40%, similar to our observations. Bergman et al. tried to find explanations in some characteristics of their MC or in a non-uniformity of their film. In our case, as for the MLC_transm test, all data were the rebinned at 1.5 mm, and this could be a reason, in some cases, of a smoothing effect, generating overestimation of the dose under the leaves (in the valleys). This effect should be more pronounced in the 2.5 mm leaves region.

Regarding the profile obtained with Acuros, the discrepancy with MC is of about 44%, and with measurements is at maximum 20%. The beam modeling of Acuros in Eclipse uses a single transmission factor, that is considered hence valid wherever in the field area and under thin or thick leaves. This approximation influences the final calculation of the alternate pattern. The approximations adopted in the MLC modeling in Eclipse seem to generate criticalities, as also pointed out by Hernanez [24].

#### Tests with dynamic MLC

##### DLG-test

**Fig. 5:**
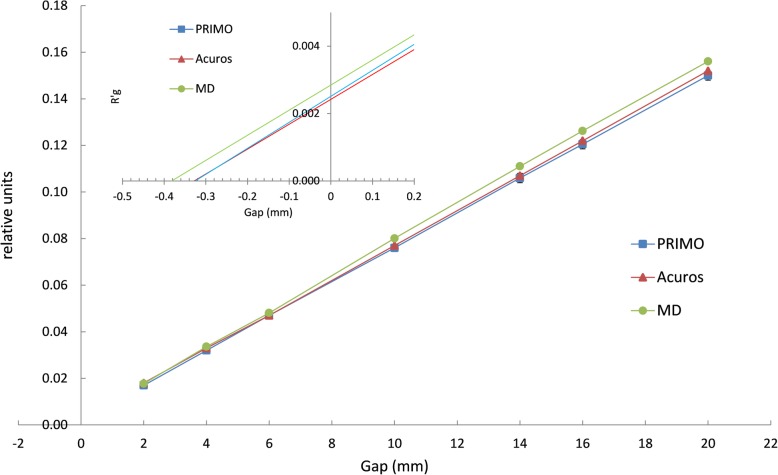
Point measurements for the different plans used in the procedure for the evaluation of DLG. Measured points are fitted with a line, whose intercept on the x axes gives the DLG value. The plot region where the intercept is visible is zoomed in the top box

1.3% average transmission was found for MC, point measurements and Acuros and subtracted to the data for DLG calculation. Results reported in Table 4 show differences within 0.008 cm among MC, measurements and Acuros. A linear fit was performed for all data series obtaining R^2^ values higher than 0.9999 in all cases (Fig. 5). The microDiamond instead (active volume 0.004, sensitivity 0.7–1.2 nC/Gy), having a better spatial resolution and a response independent from beam quality [35], obtains measurements that can be better used as a reference, though its measurement uncertainties are higher.

**Table 4:** DLG values obtained with experimental measurements, with TPS calculations and with PRIMO and relative uncertainties

	DLG (cm)
MicroDiamond	0.032 ± 0.006
PRIMO	0.033 ± 0.003
Acuros	0.038 ± 0.004

##### a-SG tests

**Fig. 6:**
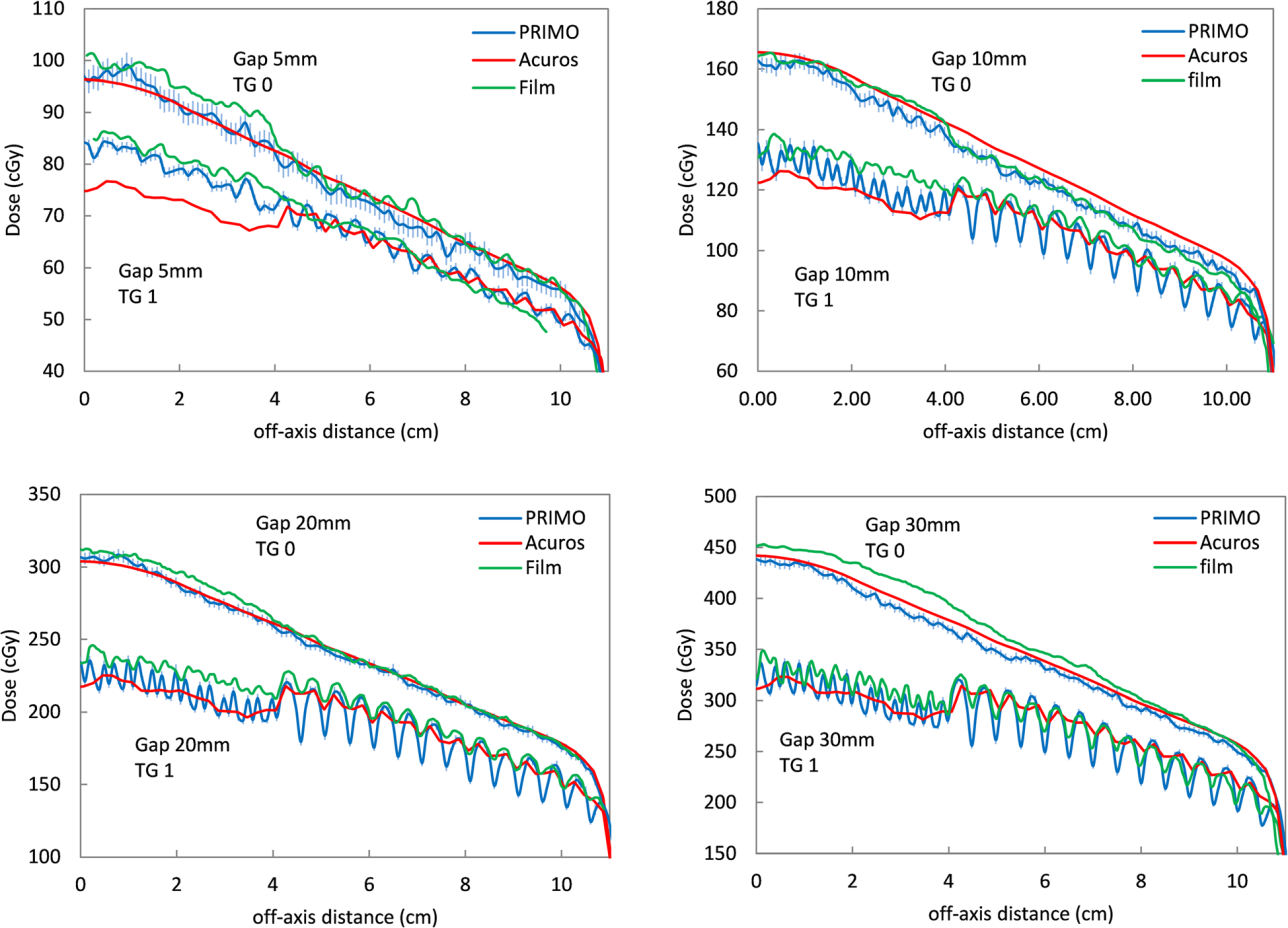
Inline dose profiles for different gap sizes and different TG fractions for PRIMO (blue), Acuros (red) and measurements with Gafchromic films (green)

Some profiles obtained with different TG fractions are reported in Fig. 6, for TG fractions 0 (all leaves aligned) and 1 (shift between adjacent leaves equal to the gap size) and three gap values (5, 10 and 20 mm).

The first effect that can be observed for all profiles is that, as the TG fraction increases, the average dose progressively decreases; this is because the TG effect is a decrement of the dose between leaves [8].

For TG 0, measured and MC-simulated dose profiles show alternate peaks and valleys due to interleaf transmission, while with Acuros this effect is not present. MC seems to have a more realistic modeling of the individual leaves, while, giving the mentioned approximation in Eclipse and explained by [24], Acuros takes into account a single transmission value for the MLC conditions. A ~ 2% dose underestimation can be observed for both MC and Acuros with respect to measurements under the thin leaves region. Under thick leaves, these differences are reduced to 0.7 and 1% for MC and Acuros, respectively.

When TG fraction is > 0, for all gap sizes, the dose profiles show an effect of average dose reduction in the 2.5 mm leaves region. This effect is more evident for increasing gap sizes. This reduction is in accordance with what found by [24]. As already observed for the TG 0 profiles, Acuros shows smaller peak-to-valley variations with respect to measurements and MC, which instead exhibits even deeper peaks and valleys. The peak-to-valley dose variations increase with increasing gaps, because the larger the gap, the more evident the TG effect, since the contribution of the MLC transmission is lower. For small gaps (i.e. 5 mm), the peak-to-valley variations are lower because the TG effect and the interleaf transmission tend to balance [24, 36].

Both MC and Acuros underestimate the dose with respect to measurements: in the 2.5 mm leaves region those differences are 4–5% and ~ 7% for MC and Acuros, respectively, in accordance with [24]. In the 5 mm leaves region the same differences are of 1.6 and 1%, respectively.

**Fig. 7:**
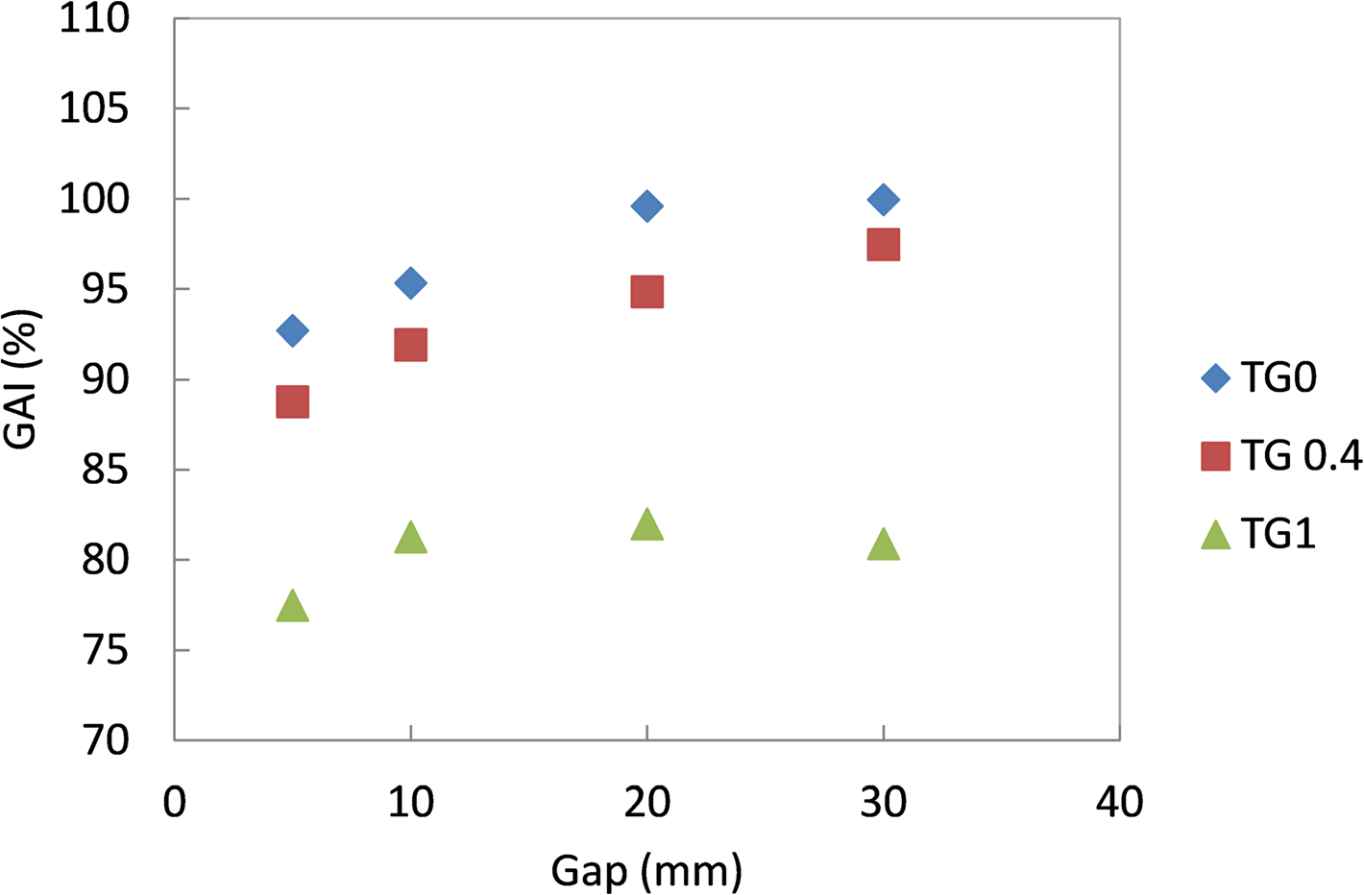
Gamma Agreement Index between dose distributions relative to the a-SG test calculated with PRIMO and Acuros inside the entire MULTIcube volume for gap sizes of 5, 10, 20 and 30 and TG fractions of 0, 0.4 and 1

A 3D analysis of the dose distributions were also evaluated for Acuros and PRIMO. The agreement between the dose distributions calculated with PRIMO and Acuros inside the entire MULTIcube volume, in terms of gamma analysis, are reported in Fig. 7 for different gap sizes and different TG fractions. This plot shows a poor agreement (below the acceptability of 95%) for any gap size when TG fraction is 1 thus indicating that the different modeling of TG effect in Acuros and PRIMO significantly affects the dose distribution. For smaller TG fraction instead, the comparison between the dose distributions give an agreement above the acceptability of 95% when TG fraction is zero. As previously described, Eclipse, as other commercially available TPSs, makes approximations in the MLC modeling and this influences the accuracy of the dose estimation. In particular the tongue and groove effect is considered by expanding the projection of the leaf and subsequently adjusting the delivered fluence in order to account for the leaf width. This two-step approximation could contribute to the observed differences with the measured/simulated dose distributions.

##### a-OSG tests

**Fig. 8:**
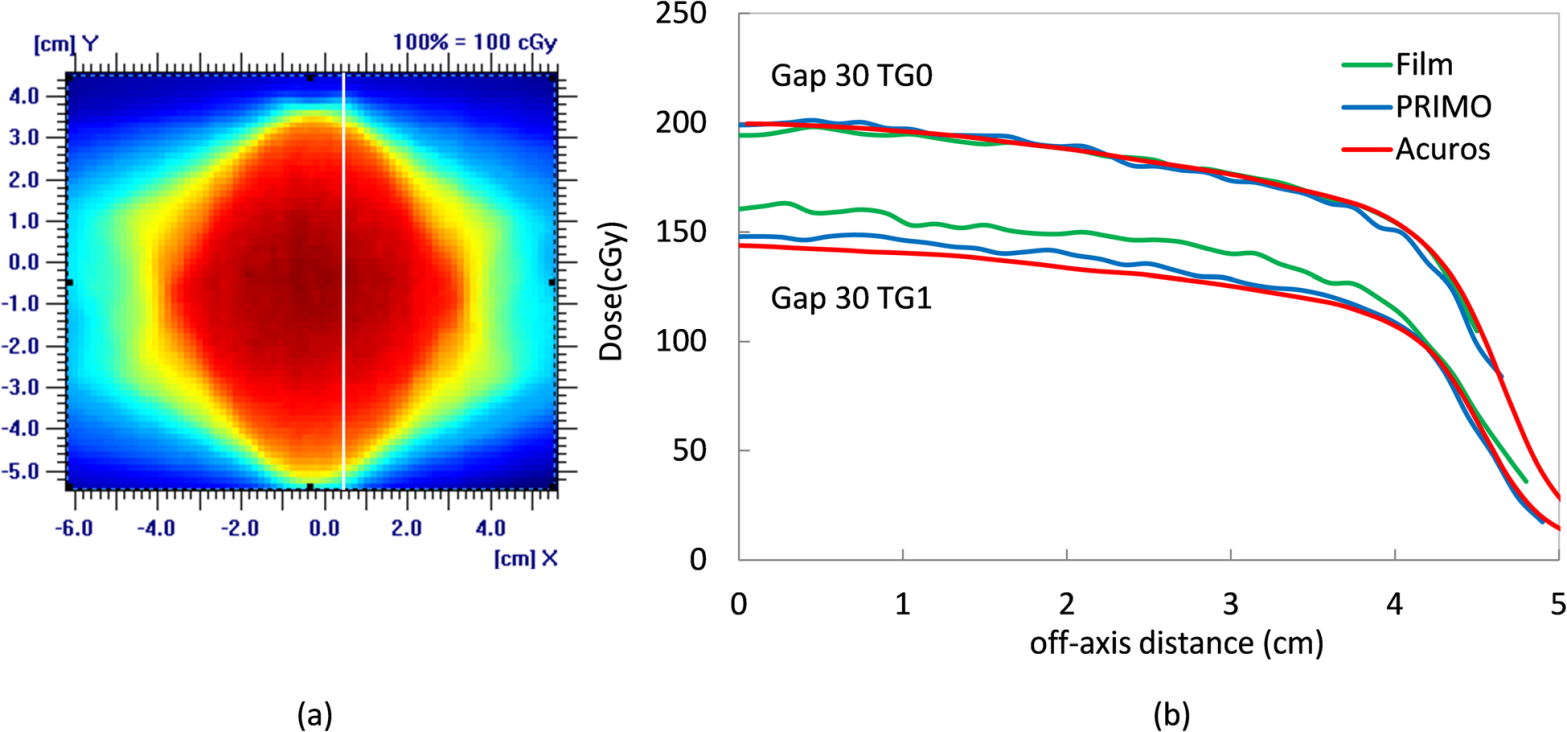
Dose distributions for the a-OSG test with the 30 mm gap for PRIMO at the isocenter level for TG fraction = 0 is illustrated in the left plane (a). The dose profiles along the straight line depicted in (b) are given for TG fractions 0 and 1

The dose distributions were analyzed through profiles at 2.5 cm off axis, as shown in Fig. 8.

Given the jaw positions in this test only the thin leaves are used and contribute to the TG effect. The profiles in Fig. 8, evaluated for gap 30 mm, show that in absence of tongue and groove (TG 0) there is a very good accordance between the profiles, with discrepancies of 0.8 and 0.5% with respect to measurements for MC and Acuros, respectively. With maximum tongue and groove effect (TG 1) the differences increase to 5 and 7% for MC and Acuros. This result is in line with [24], who found a discrepancy of 7.4% between the TPS and the film measurements. The agreement for profiles without TG shows that all the discrepancies found in the a-SG tests are reduced with the gantry rotation.

The choice of the 1.5 mm calculation grid is a known limitation for this study, particularly in the validation of dose patterns under the thin leaves of the 120-HD MLC.

### Clinical VMAT cases

The clinical plans were evaluated in terms of 3D global gamma index analysis (3%/2 mm and 2%/2 mm as dose difference and distance-to-agreement criteria) between MC in PRIMO and Acuros dose calculations, on the body structure (including the whole patients within the CT dataset) and the planning target volume PTV. The choice of the distance-to-agreement gamma criterion is consistent with the dose calculations resolution of 1.5 mm.

The gamma analysis resulted in an average gamma agreement index (GAI, defined as the percentage of the analyzed point passing the gamma criteria) for the body of 98.9 ± 0.6% for the 2%/2 mm criteria and 99.5 ± 0.2% for the 3%/2 mm, and for the PTV the GAI was 91.4 ± 0.6% and 97.7 ± 0.2% for the 2%/2 mm and 3%/2 mm criteria, respectively. The lowest GAI values for PTV were 88.2 and 97% for 2%/2 mm and 3%/2 mm criteria.

The difference in handling the MLC in PRIMO and in Eclipse, as described in the main part of this work, is only one of the reasons that could result in different dose estimations between MC and Acuros. Another important source of such differences is the different handling of the materials in the two systems, which can contribute in a twofold way. Firstly, the different material assignment according to the HU in Acuros and MC in PRIMO, as shown in Table 2, especially with the overlapping adjacent materials in Acuros, lead to different dose calculations due to different material assignment. For some materials it could be of few percent (e.g. ~ 2% between adipose and muscle [37]), higher when cartilage and bone structures are included. Secondly, the different elemental composition of the tissues in the two systems is not identical (as described in [37]), leading again to some differences in dose estimation.

The here presented results showed the importance of understanding and analyzing the parameters that could influence the dose calculation in the specific systems. From this work the MC management in PRIMO of the MLC presented better agreement with measurements than the beam source modeling for Acuros in Eclipse. In the common workflow of the clinical practice, the patients are treated with plans calculated by the TPS (Acuros in our work), and an independent dose calculation check is suggested to reduce errors induced by the dose calculation procedure. The interesting point in this flow is to understand the possible source of the discrepancies in order to properly judge results from the independent checks. However, we believe that the MC in PRIMO can be safely used for independent dose calculation checks, having proved its better management of MLC.

## Conclusions

MC simulations in the PRIMO environment were in agreement with measurements for the HD-120 MLC in a 10 MV FFF beam from a Varian EDGE linac. This result allowed to consistently compare clinical cases, showing the possible use of PRIMO as an independent dose calculation check tool.

## Data Availability

Data supporting the findings of this work are available within the article.

## Abbreviations

a-OSG: asynchronous oscillating sweeping gap test
a-SG: asynchronous sweeping gap test
CT: Computed tomography
DLG: Dosimetric leaf gap
DPM: Dose Planning Method
FFF: Flattening filter free
FWHM: Full-with half maximum
GAI: Gamma agreement index
HU: Hounsfield units
IMRT: Intensity modulated radio therapy
LBTE: Linear Boltzmann Transport Equation
MBSF: Monitor back-scatter factor
MC: Monte Carlo
MLC: Multi-leaf collimator
MU: Monitor units
OF: Output factor
PDD: Percent depth dose
PSF: Phase space files
PTV: Planning target volume
SBRT: Stereotactic body radiotherapy
SRS: Stereotactic radiosurgery
TG: Tongue and groove
TPS: Treatment planning system
VMAT: Volumetric modulated arc therapy
